# Rapid functional traits turnover in boreal dragonfly communities (Odonata)

**DOI:** 10.1038/s41598-020-71685-5

**Published:** 2020-09-21

**Authors:** Marina Schmidt Dalzochio, Eduardo Périco, Norton Dametto, Göran Sahlén

**Affiliations:** 1 Ecology and Evolution Lab, Universidade Do Vale Do Taquari, Univates, Avelino Talini Street, number 171, Universitário, Lajeado, RS 95900-000 Brazil; 2 Rydberg Laboratory for Applied Sciences, RLAS, Halmstad University, P.O. Box 823, 30118 Halmstad, Sweden

**Keywords:** Biodiversity, Climate-change ecology, Community ecology

## Abstract

All natural populations show fluctuations in space or time. This is fundamental for the maintenance of biodiversity, as it allows species to coexist. Long-term ecological studies are rare, mainly due to logistics, but studies like the one presented below recognize the dimensionality of temporal change and the ecological processes that lead to shifts in community composition over time. Here, we used three sampling occasions from a dataset spanning 20 years where dragonflies in central Sweden were monitored. Our aim was to investigate how the prevalence of ecological and biological species traits varied over time measured as Community-level Weighted Means of trait values (CWM). Most CWM values varied significantly between years. Most of the traits changed between the second and the last sampling occasion, but not between the two first ones. These changes could be linked to major changes in species abundance. Our work indicates that fundamental shifts in community structure can occur over a short time, providing environmental drivers act on species turnover. In our case, Climate change and pH levels in lakes are most likely the most important factors.

## Introduction

Fluctuations in space and/or time occur frequently in populations of species, for a multitude of reasons. There may be changes in resource availability, intra- and interspecific interactions, diseases as well as climate and landscape changes, to mention a few. Such changes can be natural (e.g., when the environment exhibits seasonal fluctuations) or anthropogenic (i.e. caused by human activities). Both abiotic and biotic factors are important in the creation of these variations^1^, although some populations remain relatively stable over long periods of time^2^. To understand how environmental change via reduced resilience affects populations, there is a need to study the involved parameters, ‘drivers’ in relation to population data^3^.

Ecologists have highlighted the importance of temporal variation in community ecology. Temporal turnover is fundamental to the maintenance of biodiversity. It allows species limited by different resources, or species that compete for the same resources, to coexist^4^. Temporal changes in species composition are caused by changes in environmental variables that favour or disfavour prominent traits of the species. Temporal studies are also important to the understanding of ecosystem functionality. The maintenance of functions over time might be promoted by a turnover between different species capable of occupying the same niche space (redundant functional species)^5^. In this case, sites with a low number of redundant functional species will lose functions more easily due to a temporal turnover in species abundances, whereas sites with a high number of redundant functional species will maintain all functions^5,6^. The understanding of these functional trends is especially important in community ecology studies.

Authors agree that a combination of traits will be characteristic of a species^7,8^, since traits shape the realized niche available to the species in its habitat. As different species have different traits, some species become abundant and others scarce, affecting the functioning of the ecosystems. Hence, a multiple trait-based approach should be more useful to identify the main drivers of change, making it possible to predict future biodiversity changes based on environmental/climate forecasts^9^.

In the present study we are looking into how species traits in an aquatic community are affected by four major environmental drivers. As many other parts of Europe, Sweden has ongoing environmental issues related to a history of acid rain deposition^10^, past and present timber harvesting^11^, changes in land use^12^ and climate change^13^. These four drivers have varied in intensity over the years, very probably affecting aquatic communities in various ways. There are also many stochastic events which affect the species composition at any given site and time^14^, but with a lower frequency in permanent than in temporary sites.

Dragonflies and damselflies (Odonata) have been suggested as ideal model organisms for monitoring ecological changes^15^ as they are sensitive to the degradation of freshwater ecosystems^9, 16^ and many species appear to be affected by environmental changes^17^. Previous studies show that dragonflies may react in many different ways to the four drivers cited above. Odonata eggs and larvae seem to be very tolerant to acid deposition^18^, while many of their predators (fishes) and prey (midges, tadpoles) are were strongly affected, causing imbalance in the food chain^19,20^. By contrast, raising pH levels by liming seems to reduce the occurrence of several common odonate species, indicating that odonate communities in limed lakes may not be natural communities^20^. Both forestry and land use change have been shown to be very detrimental to Odonata species^21–23^. Adults may have difficulties in dispersing between isolated patches and finding good breeding sites^21^.

Lastly, as all ectotherms, Odonata are dependent on temperature to regulate their activity^18^. It was recently shown that species with restricted niches were replaced by those having wider niches in response to temperature increase and higher human population density^9,24,25^. Furthermore, climate change alters precipitation regimes, decreasing winter freezing time and increasing the occurrence of heat waves. The impact of these changes varies between odonate species. This in turn will modify species interactions, phenology and development time, and ultimately alter the species composition^26^ and ecosystem functioning^27^.

Temporal fluctuations in local populations are expected to occur randomly^6^, but in a large set of communities under natural conditions, each community should vary separately from the others. The mean population fluctuation can be expected to be less variable over longer time periods, especially in a permanent habitat^14^, and react more strongly to changes in the intensity of the environmental drivers^28^. In this study we have used three sample occasions over a period of 20 years, following 30 different communities and their species populations in the same region, calculating Community Weighted Mean (CWM) of traits to be able to reduce the impact of stochastic events on individual populations or sites. Following this assumption, our first aim was to understand how a set of traits varied over the 20 years in Boreal lakes in Sweden. In specific terms, we sought to understand if the prevalence of Odonata traits had been stable over the sampled period and, if not, to identify the observed pattern. Our final question was whether the observed pattern (if any) was caused mainly by stochastic processes or determined by the impact of the four major environmental drivers: climate change, acidification, forestry and land use change.

Studies that assess the temporal dynamics of functional traits, although rare, are important because they recognize the dimensionality of temporal change and the ecological processes causing shifts in community composition over time^29^.

## Results

**Figure 1:**
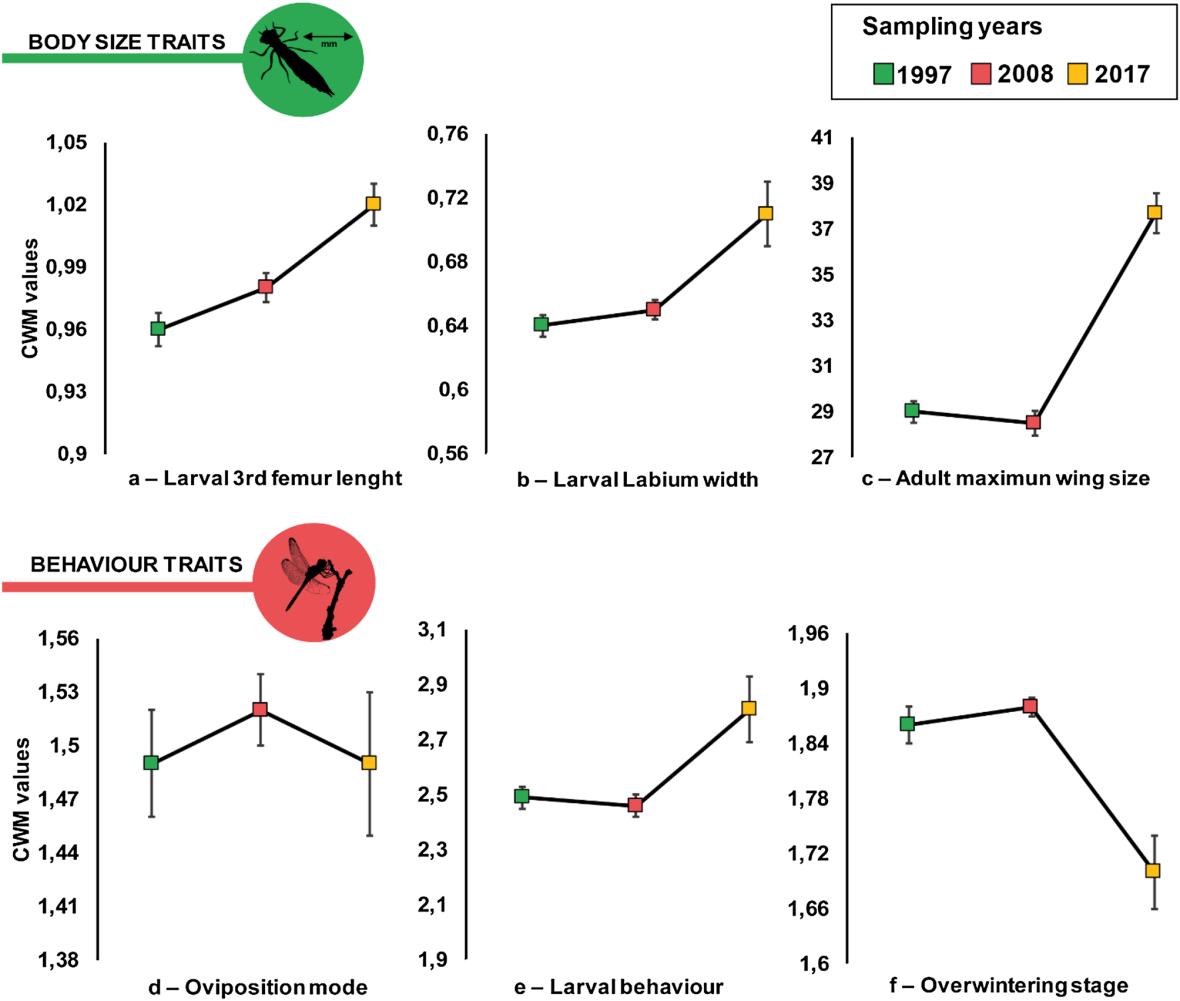
Variation in community weighted mean (CWM) of six traits related to body size and behaviour evaluated for an Odonata community of 30 lakes in the Bergslagen area, central Sweden. Mean (coloured squares) with standard error bars. Green square—1997, pink square—2008 and yellow square—2017.

**Figure 2:**
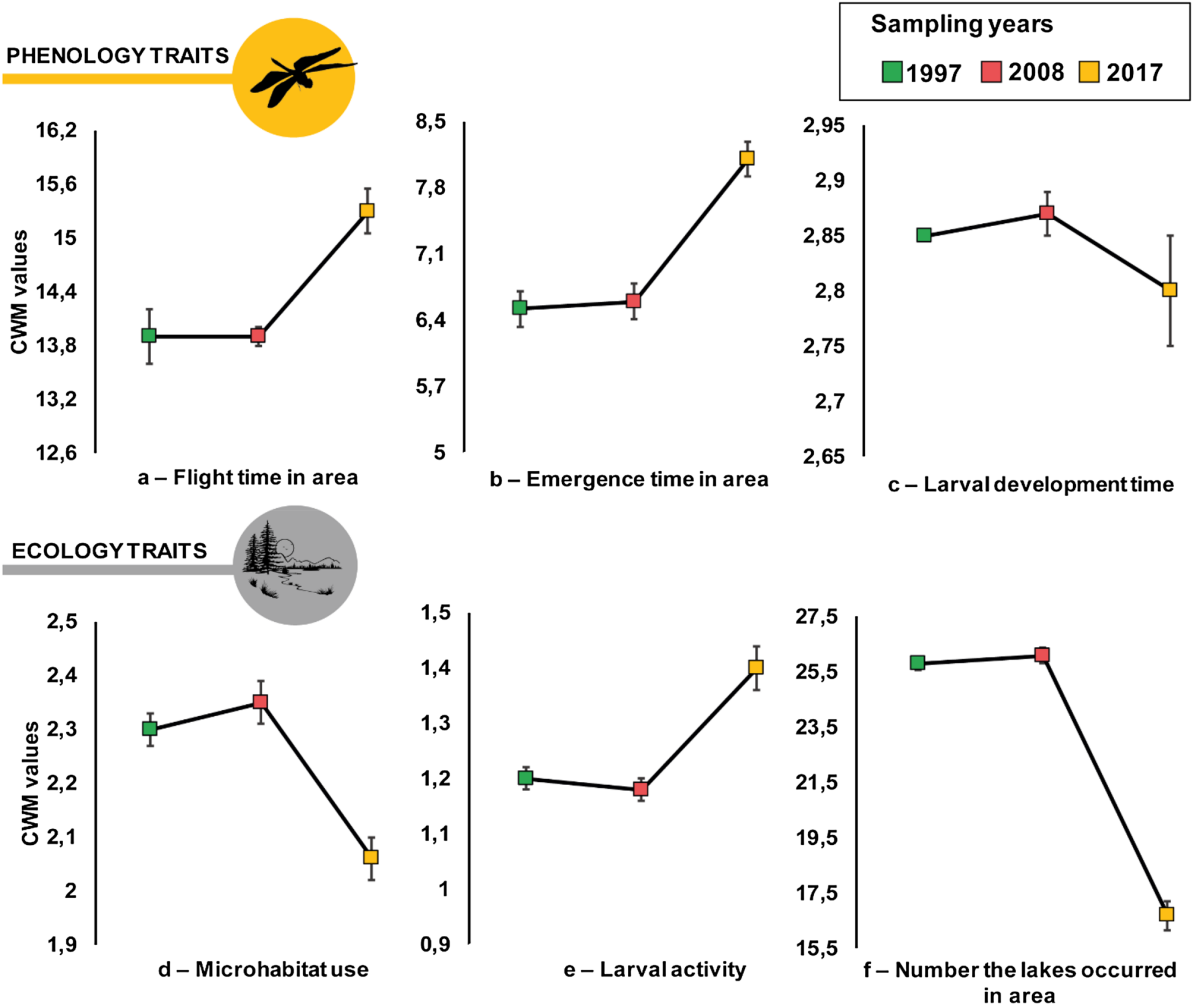
Variation in community weighted mean (CWM) of six traits related to phenology and ecology evaluated for an Odonata community of 30 lakes in the Bergslagen area, central Sweden. Mean (coloured squares) with standard error bars. Green square—1997, pink square—2008 and yellow square—2017.

The GLM results show that most of the CWM values varied significantly between years (Table 1; Figs. 1 and 2). Out of the twelve traits evaluated, seven increased from the two first sampling occasions (1997 and 2008) to the last one (2017) (Figs. 1a–c,e and 2a,b,e), three decreased between the two first sampling occasions (1997 and 2008) and the last one (2017) (Figs. 1f and 2d,f), and two did not change over time (Figs. 1d and 2c). No traits showed any significant differences between 1997 and 2008.

**Table 1:** GLM results for variation in community weighted mean (CWM) between years (1997, 2008 and 2017) in twelve traits evaluated for the Odonata community of the Bergslagen area, Sweden.

Trait	Sampling years	2008		2017	
		t-value	p-value	t-value	p-value
Morphology—Larval 3rd femur lenght	1997	1.359	0.177	− 3.761	**0.0003**
	2008	–	–	− 2.406	**0.0138**
Morphology—Larval Labium width	1997	− 0.533	0.595	− 3.742	**0.0003**
	2008	–	–	− 3.212	**0.0018**
Morphology—Adult maximun wing size	1997	− 0.582	0.562	− 9.096	**0.0001**
	2008	–	–	− 9.652	**0.0001**
Oviposition mode	1997	− 0.52	0.604	0.024	0.98
	2008	–	–	0.544	0.588
Larval behaviour	1997	− 0.382	0.704	0.837	0.405
	2008	–	–	1.218	0.226
Overwintering stage	1997	0.281	0.779	3.735	**0.003**
	2008	–	–	4.01	**0.0001**
Flight time in area	1997	0.001	0.999	− 4.266	**0.0001**
	2008	–	–	− 4.266	**0.0001**
Emergence time in area	1997	0.314	0.754	− 5.503	**0.0001**
	2008	–	–	− 5.197	**0.0001**
Larval development time	1997	− 0.364	0.716	− 3.003	**0.003**
	2008	–	–	− 3.363	**0.0045**
Microhabitat use	1997	− 0.87	0.38	3.979	**0.0001**
	2008	–	–	4.841	**0.0001**
Larval activity	1997	− 0.455	0.65	− 4.787	**0.0001**
	2008	–	–	− 5.235	**0.0001**
Distribution in the area	1997	− 0.338	0.736	14.873	**0.0001**
	2008	–	–	15.174	**0.0001**

Bold p-values are significant.

All size-related traits varied in the same way: 3rd femur length and labium width of larvae as well as maximum wing length were much higher in the 2017 community than in 1997 and 2008 (Table 2; Fig. 1a–c). CWM for flight time showed a predominance of species occurring more than 15 weeks as adults in 2017. In 1997 and 2008, by contrast, species with an adult lifespan of 8 to 12 weeks were predominant. Likewise, species with an emergence period exceeding 8 weeks were significantly more abundant in 2017 than in 1997 and 2008, when the predominant species had an average emergence period of 6 weeks.

**Table 2:** Mean and standard error for variation in community weighted mean (CWM) between years (1997, 2008 and 2017) in twelve traits evaluated for the Odonata community of the Bergslagen area, Sweden.

Traits/year	1997	2008	2017
Larval 3rd femur lenght	0.96 ± 0.008	0.98 ± 0.007	1.02 ± 0.01
Larval Labium width	0.64 ± 0.007	0.65 ± 0.006	0.71 ± 0.02
Adult maximun wing size	29.0 ± 0.47	28.5 ± 0.56	37.7 ± 0.88
Oviposition mode	1.49 ± 0.03	1.52 ± 0.02	1.49 ± 0.04
Larval development time	2.85 ± 0.0	2.87 ± 0.02	2.80 ± 0.05
Larval behaviour	2.49 ± 0.04	2.46 ± 0.04	2.81 ± 0.12
Overwintering stage	1.86 ± 0.02	1.88 ± 0.01	1.70 ± 0.04
Flight time in area	13.9 ± 0.30	13.9 ± 0.11	15.3 ± 0.25
Emergence time in area	6.52 ± 0.19	6.60 ± 0.19	8.10 ± 0.18
Microhabitat use	2.30 ± 0.03	2.35 ± 0.04	2.06 ± 0.04
Larval activity	1.20 ± 0.02	1.18 ± 0.02	1.40 ± 0.04
Distribution in the area	25.8 ± 0.22	26.1 ± 0.29	16.7 ± 0.52

With regard to larval development time, we observed that species which take more than two years to develop have decreased in abundance, bringing the CWM value down. However, there was no statistical change in the trait patterns, which remained the same over the years. In 2017, the mean CWM value was 2.80, while for 1997 and 2008 this number was 2.85 and 2.87 respectively (Table 2; Fig. 2a–c).

**Figure 3:**
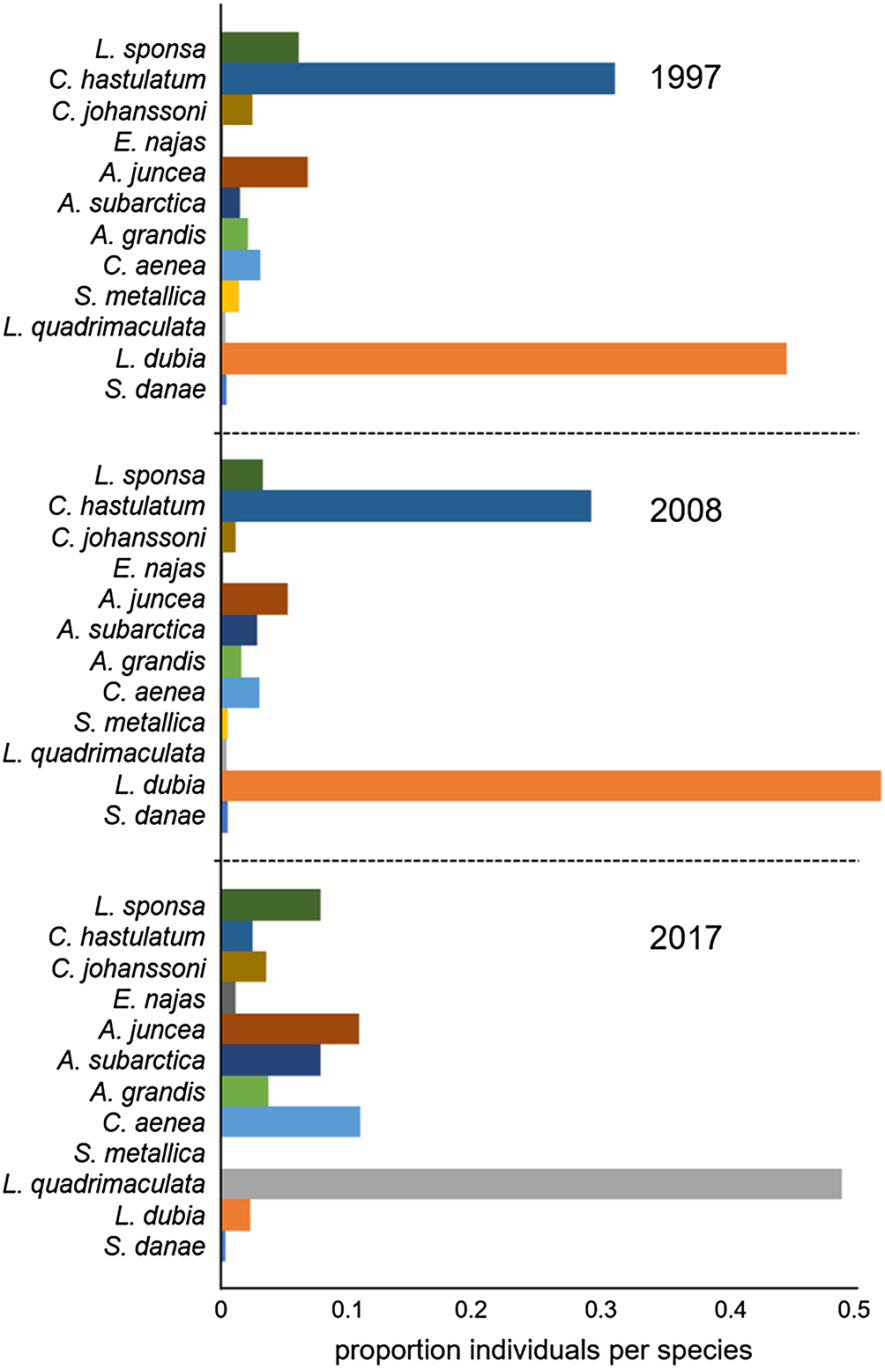
Proportion of individuals per species for sampling years (1997, 2008 and 2017) in an Odonata community of 30 lakes in the Bergslagen area, central Sweden.

For the traits oviposition mode and larval behaviour, the GLM analysis did not show any differences between sampling years (Fig. 1d,e). However, the CWM value for overwintering was significantly lower in 2017 (Table 2; Fig. 1f). Here, as in the case of larval development time, there was no change in the pattern of the trait. A decrease in abundance of species overwintering as larvae (code 2) or both as egg and larvae (code 3) could be noticed between the years. *Aeshna grandis* (Linnaeus, 1758), for example, (code 3) decreases in abundance from 159 to 88 individuals. Also *Coenagrion hastulatum* (Charpentier, 1825) and *Leucorrhinia dubia* (Vander Linden, 1825) (code 2) decreased in abundance between years (Fig. 3; for details, see Supplementary material).

We observed a decrease in traits prevalence for larval microhabitat use in 2017 compared to the earlier samples (Table 2; Fig. 2d). Species that use the water column (code 2) or live near the water surface (code 3) decreased in abundance between the years. *Somatoclora metallica* (Vander Linden, 1825), for example, uses the water column (intermediate—code 2) and decreased in abundance from 106 to 2 individuals (Fig. 3). For the larval activity trait, species with high activity were more abundant in 2017 than in 1997 and 2008 (Table 2; Fig. 2e). Also, for occurrence in the area, CWM was significantly lower in 2017 compared to 1997 and 2008 (Table 2; Fig. 2f). A majority of the species reduced their distribution in the lakes sampled. For example, *S. metallica* was present in 11 lakes in 2008, but in 2017 it was only present in a single lake (for details, see Supplementary material).

Comparing patterns of occurrence in the area, we noted that the most pronounced changes occurred between 2008 and 2017. *Coenagrion hastulatum* and *Leucorrhinia dubia*, which were very common during 1997 and 2008 decreased to very small numbers, whereas *Libellula quadrimaculata* Linnaeus, 1758, increased (Fig. 3). We also noted that the three species of the genus *Aeshna* Fabricius, as well as *Cordulia aenea* (Linnaeus, 1758), have at least doubled in numbers between 2008 and 2017.

## Discussion

In this study we were able to show how changes in multiple traits in a community of Odonata switched rapidly during the latter half (nine years) of the 20-year study period. This is the first assessment using a multiple-trait based approach to evaluate long-time changes in an Odonata community. To our knowledge, other investigations attempting to identify functional changes are short-term studies^9,25^ restricted to phenological attributes.

**Figure 4:**
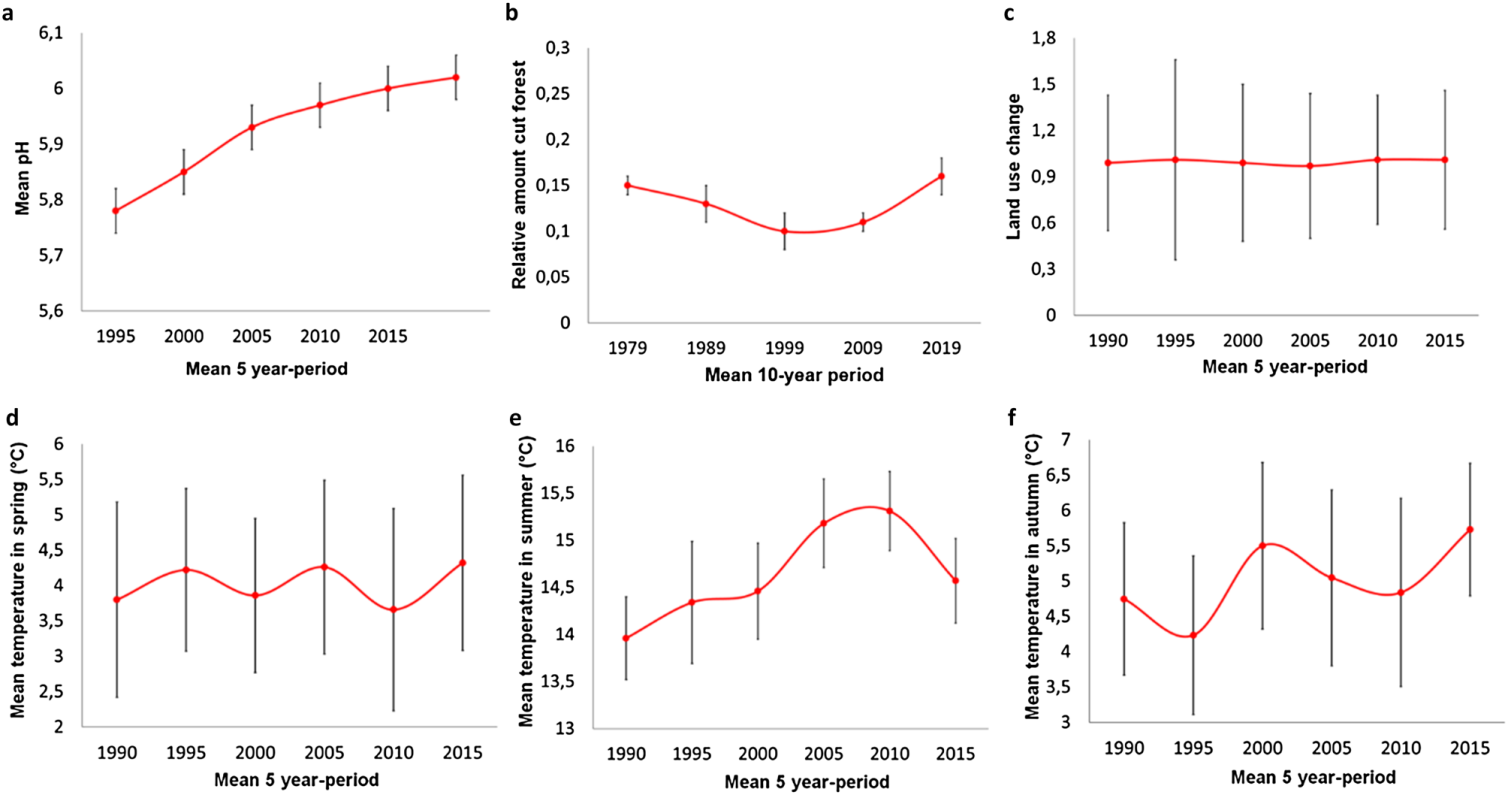
Historical data of major environmental drivers evaluated for five- or ten-year periods in the Bergslagen area: Potential of hydrogen (pH) (**a**); Relative amount of forestry activities within 500 m of shoreline (**b**); Relative amount of land use change (**c**); Mean temperature at onset of spring (**d**), summer (**e**) and autumn (**f**).

In Sweden, several authors report effects of climate change^24,30^, forestry^21^, land use change^21^ or acidification^19,20,31^ on aquatic community structure; especially for Odonata, and mainly with regard to species composition. Our results show that the prevalence of traits was different between years, but we did not observe any significant change in Odonata trait composition during the first sample interval (1997 to 2008, Figs. 1 and 2). Instead, the changes were generally abrupt and occurred between 2008 and 2017. This is consistent with big changes in the environment observed from 2005 to 2015 (Fig. 4). We therefore believe that the recorded CWM changes are to a major part caused by the four major environmental drivers – an assumption that is discussed below.

Our analysis revealed that in 1997 and 2008, the odonate assemblages were dominated by smaller species with shorter adult life span and emergence time (spring species^32,33^), such as *C. hastulatum* and *L. dubia*. In 2017, the community was instead dominated by large species with longer adult life spans and a longer emergence period (summer species^32,33^), like *A. juncea* (Fig. 3). *L. quadrimaculata* is a spring species with a long emergence period which was also becoming abundant in 2017. On the other hand, although we observe statistically different CWM values between the first sampling years (1997 and 2008) and the last (2017), the status of traits such as overwintering stage, larval development time, microhabitat use and larval activity (cf., Table 3) remained constant throughout the years. For example, the CWM of microhabitat use in 2017 is 2.06, which indicates a dominance of species that use the water column. The value is similar for the years 1997 (2.30) and 2008 (2.35). The conclusion seems to be that body size, larval development and emergence time is related to the change in environmental drivers^34^, in our case primarily temperature^3,35^. Following the general rules of ectothermic organisms, we might expect that warmer temperatures would give shorter development time. A shorter development time, in turn, often results in smaller individuals^36^, as species become ready for emergence earlier^37^. These patterns often apply to species with simple life cycles^38^, but for Odonata, where both the number of larval instars and the development time are variable, we expect many deviations from this rule. Some earlier studies^39^ show that the growth rate of Odonata larvae reaches maximum at a mean temperature of 20 °C, growing in a logistic way from 12 °C to 20 °C. At temperatures above 25 °C, both the growth rate and the body size decreased. Early instars of *Coenagrion puella* (Linnaeus, 1758) reduced their moult intervals if the temperature rose from 12 to 16 degrees, but with no costs in body size. In Sweden, mean summer temperatures have indeed increased since 1985, but they have not yet reached a higher summer mean than ~ 20 °C (Fig. 4d). This means that until now, the rising temperature should result in an increased growth rate, or rather a shift toward fast-growing species, although this is very probably variable^38^. Looking at the proportion of species present at the sampling occasions (Fig. 3), we note that proportions are comparable between 1997 and 2008, while the dominant species on these occasions (*C. hastulatum* and *L. dubia*) are replaced by *L. quadrimaculata* in 2017. On this occasion, we also note a higher proportion of *Aeshna* and *Cordulia* species. The former two species grow to a smaller size than the latter, and the development time is often 1–3 years in southern Sweden. *L. quadrimaculata* grows fast^40^, and *Aeshna* grow slower (2–4 years)^32,41^. In our area, species with a medium fast life cycle have been replaced by slow-growing and fast-growing species, indicating a complex relationship where several additional factors are probably involved. There have also been well-documented and considerable temporal shifts in the emergence period of Odonata^42^, and evidence that dragonflies and damselflies have advanced their flight dates over recent years due to complex effects of changing temperature regimes^43^. It is, thus, possible that life cycle alterations caused by these temporal changes might be part of the explanation for the observed changes in abundance.

**Table 3:** Functional traits evaluated for the Odonata community in the Bergslagen area in the years (1997, 2008 and 2017).

Trait	status	code	Source
Morphology	Larval 3rd femur length	Continous	Personal observations
	Larval Labium width	Continous	Personal observations
	Adult maximum wing size	Continous	^73^
Oviposition	Exophytic	1	^9^
	Endophytic	2	
Larval development time	1 year	1	^31,74–76^, and Sahlén personal observations
	2 years	2	
	Over 2 years	3	
Overwintering stage	Egg	1	^9^
	Larvae	2	
	Both	3	
Flight time in area	Number of weeks	Continuous	Swedish Species Observation System (www.artportalen.se), local area 1996–2017
Emergence time in area	Number of weeks	Continuous	Swedish Species Observation System (www.artportalen.se), local area 1996–2017
Larval behaviour	Clasper type 1	1	^33^ and Sahlén personal observations
	Clasper type 2	2	
	Sprawler	3	
Microhabitat use	Bottom	1	^50^
	Intermediate	2	
	Surface	3	
Larval activity	Low	1	^50^
	High	2	
Distribution in the area	Number of lakes occupied in area	Continuous	Personal observations

Some studies show that the variation in emergence time can probably be attributed to environmental conditions such as temperature^44,45^, but also to habitat structure or food availability. The changes caused by acid deposition affect aquatic organisms in different ways^19^. It is known that dragonflies show a high tolerance to acidity^20^. A decrease in pH values does, however, increase water transparency, thus creating changes in habitat structure, competition and predation rates. Such changes might, e.g., benefit fast-growing species, provided that these species are able to attain a sufficient size to effectively feed on other species within a certain time window^46^. Other studies do, however, suggest that smaller species may easily avoid predation by reducing their activity, or by adopting other defensive behaviours^47^. In our case we noted an earlier emergence and, consequently, an extension of the flight period. If species advance their phenology (i.e., become active earlier in the season), this may cause a temporal mismatch affecting the synchrony of key activities with the supply of food or habitat, which can lead to population crashes or even extinctions^48^. For generalist predators like the Odonata, such mismatches will, however, probably have only a marginal effect on individual fitness.

At the third sampling time, species were to a higher extent using the benthic part of the littoral. Although warmer weather may decrease the time of freezing over and match temperatures from surface and bottom, warmer weather also causes more intense rainfall and harder winds, as has been manifested during the past decade^49^. Warming can further reduce the period of winter stagnation in lakes (if the lakes don’t freeze) and thereby induce a possible winter circulation of the whole water volume. This would favour species that are more temperature tolerant^45^. Whether any of the species seemingly favoured in 2017 (Fig. 4) belongs to this group is not clear, however, as several Odonata seem to have the same temperature optimum^35^, possibly also when living at high latitudes.

Species were more active in 2017 than in 1997 and 2008. These results may reflect recovery of the lakes due to less acid deposition. Most insectivorous fish decrease their abundance at a low pH, allowing the abundance of slow-lifestyle species to increase. The larvae of species with a slow lifestyle (low activity^50^) are shown to be susceptible to fish predation^51^, while active species are more efficient in their antipredator behaviour (swimming and lamellae loss). It is, however, not clear whether there is any increase in the fish population in our study area. Our localities are not sampled for fish, and lakes within the area which are regularly monitored show no trend toward increased fish abundance^52^.

Our results also indicate a prevalence of species that overwinter in the egg stage in the last sampling year. There is a possible phenology effect here, as higher temperatures may cause eggs to hatch earlier in the year^42^. The hatching of eggs is also to a large extent regulated by day length, especially at high latitudes^41^. Some species have an obligatory overwintering as eggs, while others hatch in late summer so that the larvae may reach a sufficient size (winter critical size^42^) to survive the starvation of a cold autumn followed by an overwintering^44,53^. In our area, the genera *Aeshna, Lestes* and *Sympetrum* overwinter as eggs, while *Somatochlora* can facultatively overwinter in the egg stage^32^. One explanation to our observed pattern is therefore that members of some these genera have become more common in later years (cf., Fig. 3). It is also known that higher temperatures could delay the induction of diapause, thereby causing increased (winter) mortality, as diapause is used to avoid periods of low energy availability^54^. The depletion of stored nutrient resources, caused by a higher metabolic rate, are more detrimental to larvae than to eggs. This would directly affect the survival and fecundity of the adults, compromising the persistence of populations^55^.

In our region, many species occurred in fewer lakes in the last sample than in the two earlier samples. At first, we expected that trait 12 (occurrence in area) varied with body size, which is known to be a good trait for dispersal^27^. However, there is no strong support that range shifts are linked to morphological traits such as body size^56^. In our case, the explanation seems to be complicated. We see a sudden shift from a species assembly which is relatively stable from 1997 to 2008, whereas seven out of twelve traits suddenly increased in the assembly between 2008 and 2017. Furthermore, two traits were not affected and three decreased, including regional occurrence in number of lakes. Although two of the changing traits altered more gradually (the size related traits), all others changed abruptly. Climate change can cause a rapid species turnover in regional odonate communities over a 10-year period^24^. Flenner and Sahlén^24^ noted that the influx of southern species was only marginal; a small set of species appearing in only a few of the studied lake communities. But even with a small regional occurrence, a new species may cause changes in the abundance and survival ability of resident species^35^.

In this paper, we have assessed only a handful of environmental drivers which we deem to be the most important ones in our area. Of these, pH in lakes and temperature show a clear change over the study period, and they are doubtless important reasons for the patterns observed. It is well known, however, that at least in Europe, many species of Odonata have declined from the mid 20th century and onward due to agricultural intensification, with changed hydrology, fertilizers, pesticides and pollution as important components^57^. We also recognize that it is difficult to interpret any pattern derived from only three measurements over 20 years. During the long time between sampling occasions, stochastic events have undoubtedly been both common and in some cases comprehensive^58,59^. Following a single population of a single species in this way would yield very limited information, as some species have very fluctuating populations while others are stable^2^, but both environmental drivers and other events would easily change the population size. An example is the long-time study of the genus *Sympetrum*, where temperature changes have been shown to induce different intervals of population cyclicity^60^. Looking at our traits in relation to the environmental drivers (Figs. 3, 4), there is a clear correspondence between temperature change and pH on one side, and the changing traits on the other side. If the trait changes were due to stochastic reason, we would expect a similar amount of change during the two intervals, 1997–2008 and 2008–2017, respectively. Our pattern derived from 12 populations in 30 lakes is clearly not stochastic. Instead, we see that the traits are affected differently, and possibly by different environmental drivers (Fig. 4). The picture is complicated, but clearly climate, followed by pH, has the largest impact on the species assembly.

## Conclusions

Our trait-based approach highlighted significant changes in morphology, phenology, activity rates, microhabitat use and occurrence between the studied years (Figs. 1 and 2). If the suggested drivers (foremost climate change or acid rain deposition) are the reasons behind the array of changes observed here, they might have caused more marked changes between 2010 and 2015, forcing our community in Bergslagen to change considerably between 2008 and 2017. The effects are strong and affect many ecological parameters in the community. Although we were able to observe these changes taking place during a 9-year period, we cannot tell whether the community we see today has stabilized, or if the changes are continuing. We believe that further research is needed to evaluate the exact nature of the changes, and what the new community structure means to a lake community from a broader, food-web based ecological perspective.

## Methods

### Study area

**Figure 5:**
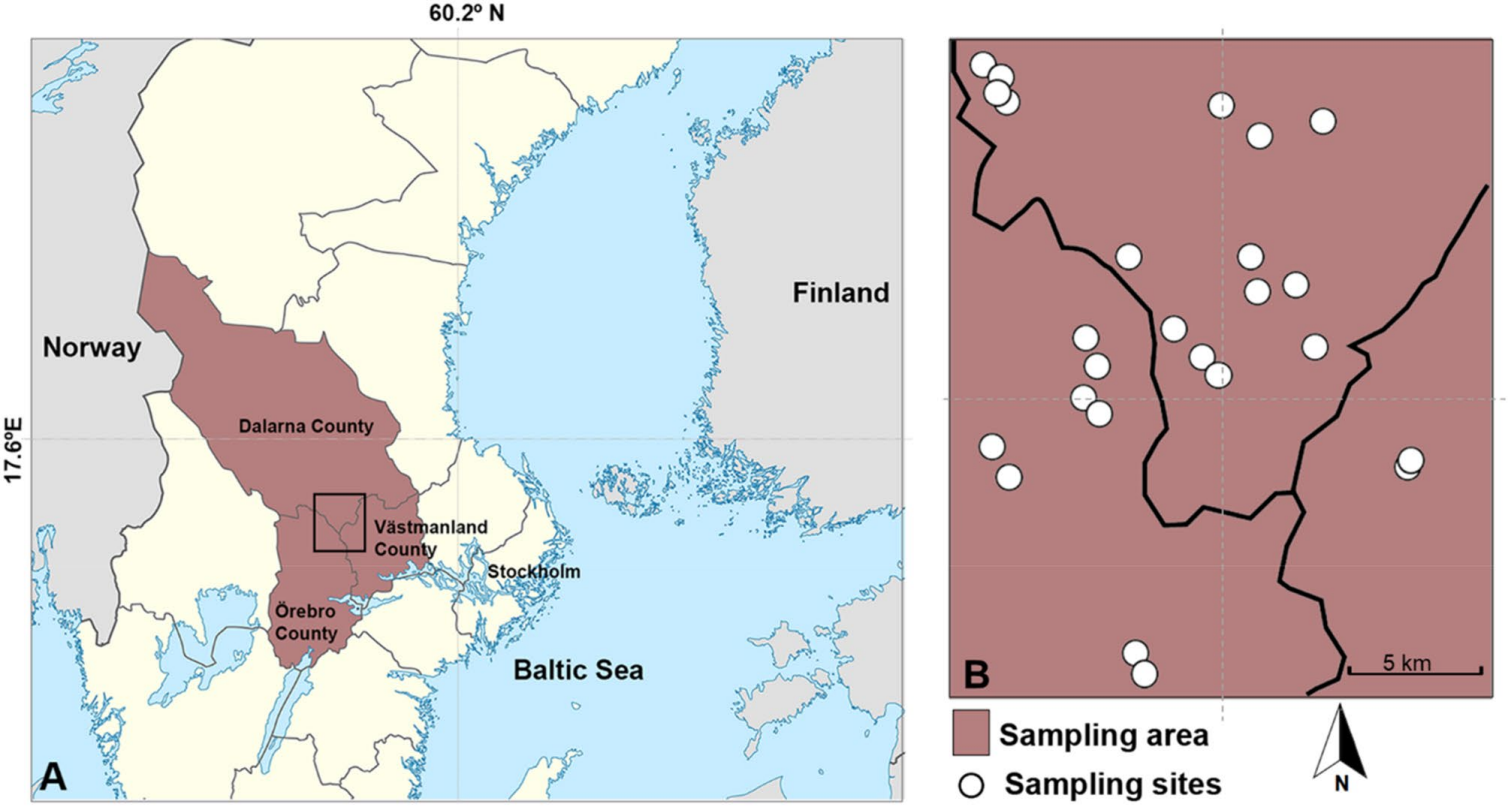
Map of Sweden with location of the Bergslagen area (black square at the juncture of the administrative regions of Dalarnas, Örebro, and Västmanlands län) (**A**) and detail of the 30 sampled lakes (white circles) within the area (**B**). Sweden map generated by MSD in Adobe Photoshop (www.adobe.com), based on map of Sweden in Wikipedia (https://upload.wikimedia.org/wikipedia/commons/d/df/Sweden_location_map.svg) which is used and adapted under Creative Commons Attribution-Share Alike 3.0 Unported license (https://creativecommons.org/licenses/by-sa/3.0/deed.en). Insert map drawn by GS adapted from Al Jawaheri & Sahlén^20^.

Our study sites are situated in the Bergslagen region, Central Sweden, at the juncture of the administrative regions of Dalarnas, Örebro, and Västmanlands län (c. 60° N, 15° 30′ E; Fig. 5). The region is characterized by a warm summer-humid continental climate (Dfb Köppen) with an annual precipitation of more than 500 mm. The average temperature is about 12.9 °C from May to September and about − 0.3 °C from October to April^49^. Snow and ice are present during 75 to 156 days, normally from late December to March (2005–2016)^49^. The elevation ranges from 150 to 300 m.

### Field methods

Our dataset is derived from the monitoring of dragonflies in lakes in central Sweden, using three sampling occasions over 20 years (1997, 2008 and 2017). Monitoring methods were devised by GS for an ongoing, decade-wise biodiversity survey. Methods and sampling effort were comparable between years, albeit performed by different people. Odonate assemblages were surveyed at 30 lakes during July and August of 1997, 2008 and 2017. This time of year (along with early spring) is the best time to sample odonate larvae at high latitudes, as many of the species (especially those in the instars relatively close to emergence) have entered long day diapause^32,41^. During this period many individuals accumulate in the penultimate instar (F-1) in preparation for overwintering in the final instar (F-0). In the case of univoltine species, most individuals will already be emerged or close to emerging, but in *Lestes sponsa* and *Sympetrum danae* the emergence period is relatively long, and hence these two species will also be present in the samples.

At each site, Odonata larvae were sampled with a standard water net (22 cm wide, 1.5 mm sieve^16^), taking 30–40 random ‘nettings’ near the shore and in all different littoral habitats occurring in the lake. Lake shores were similar with (a) some small parts dominated by rocks, sand and gravel, (b) some parts with *Sphagnum* vegetation reaching the shoreline with stands of *Carex* and *Nuphar* outside, (c) some parts dominated by dense *Carex* and *Menyanthes* and (d) some small parts with stands of *Phragmites*. Each ‘netting’ was composed of three consecutive sweeps, approximately 1 m long and at the same place to capture escaping individuals. The method has been extensively used in many Odonata studies in Scandinavia^16^. All larvae were preserved in 80% ethanol and identified using the standard key for the area^32^. All voucher specimens are stored in the RLAS laboratory at Halmstad University, Halmstad, Sweden. In two previous publications, the senior author (GS) has used the records of species occurrences (presence/absence) at the sites in 1997 to analyse biodiversity indicators^61^, and the data from three sampling years has been used as part of the dataset for investigating occupancy frequency distribution patterns in odonate species assemblies^62^. Here we use the data in a different way.

No collection permit is needed for aquatic invertebrates in the studied area.

### Environmental drivers

i. *Acidification and countermeasures*: From the 1960s until the mid-1990s, Swedish lakes suffered from acidification caused by large amounts of sulphur emitted from fossil fuel combustion and industrial process^10,20^. Acidification of surface waters has decreased substantially since then, and many acidified lakes show clear signs of recovery^10,63^, either from natural processes or because of extensive national liming programs^20^. Data from MAGIC biblioteket sjöar^64^, modelled for the lakes of the area, indicate a significant increase in pH values during last 25 years (Fig. 4a). This is confirmed by a comparison with current pH values measured in three lakes in the area by the county administrative board of Örebro.
ii. *Forestry intensity:* Being an important pillar of Swedish economy, production forests cover about half of Sweden’s territory. Although the planted area remains almost the same, clear cutting has increased slightly during the last decades^65^, causing continuous biodiversity loss. The forests in the region are largely owned by the company Sveaskog. The amount of forestry activities (thinning and clear-cutting in the area up to 500 m from the shoreline of 28 of the 30 lakes) have been stable over the past 30 years (Sveaskog, Fredrik Gunnarsson, pers. comm.; Fig. 4b).
iii. *Climate change:* Climate change affects many ecosystems on earth^24,48^. According to open data^49^, the region registered historically low values of snow cover and snow depth during the last 10 years. In addition, annual global radiation and air temperature increased. Using the open data^49^ collected at the weather station Kloten in the centre of our study, area we compared mean air temperatures of Spring, Summer and Autumn (from March to November) over the years 1985–2016. Considering that there is no larval activity at water temperatures below 5 °C, the winter months (Dec–Feb) were excluded^32^. Temperatures changed considerably during the last 5 years of the study (Fig. 4c,d).
iv. *Land cover change:* Statistics Sweden^66^ shows that agricultural areas have decreased in size from the early 1900s up to 2015, while grassland and pastures have been stable and even increased moderately since the late 1970s. During the past few decades, however, city expansion has often occurred also in forested areas. In our region, historical data^66^ shows only minor changes in the number of hectares of arable, grazing and productive forest land during the past 25 years (Fig. 4e). The number of new buildings over the past 25 years has also changed very little from 2000 to today (Fig. 4f).

### Trait selection and measurement

We selected twelve ecological/biological traits, based on previous traits change papers^9,42,48,67,68^: 3rd femur length, labium width, maximum wing size, oviposition type, larval behaviour type, larval development time, overwintering stage, flight time in the area, emergence time in the area, microhabitat use by larvae, larval activity and, finally, occurrence in the area (regional occurrence, as a measurement of how common or rare the species is). The traits were divided into 24 categories (Table 3); some of which were continuous, others categorical. Most of the trait information was obtained from publications or open databases as listed in Table 3. For the size-related traits of larvae (3rd femur length and labium width), we randomly selected 10 individuals from each year and corrected for intraguild larval size differences by dividing the measure value by head width.

### Species selection

We selected twelve species, which were well represented in all three sampling years: *Aeshna grandis* (Linnaeus, 1758), *A. juncea* (Linnaeus, 1758), *A. subarctica* Walker, 1908, *Coenagrion hastulatum* (Charpentier, 1825), *C. johanssoni* (Wallengren, 1894), *Cordulia aenea* (Linnaeus, 1758), *Erythromma najas* (Hansemann, 1823), *Lestes sponsa* (Hansemann, 1823), *Leucorrhinia dubia* (Vander Linden, 1825), *Libellula quadrimaculata* Linnaeus, 1758, *Somatoclora metallica* (Vander Linden, 1825) and *Sympetrum danae* (Sulzer, 1776). This species selection is also taxonomically representative, as the species belong to five families and both suborders (Zygoptera and Anisoptera) of Odonata. All selected species are widespread in Sweden. Many are common also over large geographical areas in Europe and the Palearctic, or have a Holarctic distribution.

### Community-level weighted means of trait values (CWM)

To assess the variation in trait composition over the years, we calculated a community weighted mean index (CWM)^69^ using package FD^70^ in R, version 3.2.4^71^. This index represents the relative abundance of each trait within each studied year, and was calculated by correlating the species trait matrix with the species abundance matrix^72^. For this study, CWM is defined as: $CWM={\sum }_{i=1}^{n}{p}_{i } \times {traits}_{i}$, where ${p}_{i}$ the relative contribution of species $i$ to the community, and ${traits}_{i,}$ is the trait value of species *i*^69^. The relative contribution *p*_*i*_ for each species was derived from the abundance (number of individuals) in the region (30 localities combined) for each sampling year. We compared these between years (1997, 2008 and 2017) using Generalized Linear Models (GLM) with Gamma family distribution, as our variables are both categorical and continuous, with the CWM values as the response variable and year as the explanatory variable. This analysis was performed using GLM function in R version 3.2.4^71^. The model was not evaluated, as there was only a single response variable, comparing each trait between years.

## Supplementary information

Supplementary file1
